# Preoperative CT or PET/CT to Assess Pelvic and Para-Aortic Lymph Node Status in Epithelial Ovarian Cancer? A Systematic Review and Meta-Analysis

**DOI:** 10.3390/diagnostics11101748

**Published:** 2021-09-23

**Authors:** Camille Mimoun, Roman Rouzier, Jean Louis Benifla, Arnaud Fauconnier, Cyrille Huchon

**Affiliations:** 1 Department of Gynecology and Obstetrics, Lariboisiere Hospital, University of Paris, 75010 Paris, France; jlbenifla@gmail.com (J.L.B.); cyrille.huchon@aphp.fr (C.H.); 2 Research Unit EA 7285 “Risk and Safety in Clinical Medicine for Women and Perinatal Health”, University of Versailles Saint-Quentin (UVSQ), 78180 Poissy, France; Arnaud.Fauconnier@ght-yvelinesnord.fr; 3 Department of Surgical Oncology, Curie Institute, 92210 Paris, France; roman.rouzier@curie.fr; 4 INSERM U900 STAMPM Team, 92210 Saint Cloud, France; 5 Department of Gynecology, Poissy-St Germain Hospital, 78300 Poissy, France

**Keywords:** CT, PET/CT, lymph node metastasis, epithelial ovarian cancer, diagnostic accuracy, meta-analysis

## Abstract

Background: In advanced epithelial ovarian cancer (EOC), the LION trial restricted lymphadenectomy indication to patients with suspect lymph nodes before and during surgery. Preoperative imaging is used to assess lymph node status, and particularly CT and PET/CT. The aim of this systematic review and meta-analysis was to evaluate the diagnostic accuracy of preoperative CT and PET/CT to detect lymph node metastasis (LNM) in patients with EOC; Methods: Databases were searched from January 1990 to May 2019 for studies that evaluated the diagnostic accuracy of preoperative CT and PET/CT to detect LNM in patients with EOC with histology as the gold standard. Pooled diagnostic accuracy was calculated using bivariate random-effects models and hierarchical summary receiver operating curve (HSROC). This study is registered with PROSPERO number CRD42020179214; Results: A total of five studies were included in the meta-analysis: four articles concerned preoperative CT and four articles concerned preoperative PET/CT, involving 106 and 138 patients, respectively. For preoperative CT, pooled sensitivity was 0.47 95% CI [0.20–0.76], pooled specificity was 0.99 95% CI [0.75–1.00] and area under the curve (AUC) of the HSROC was 0.91 95% CI [0.88–0.93]. For preoperative PET/CT, pooled sensitivity was 0.81 95% CI [0.61–0.92], pooled specificity was 0.96 95% CI [0.91–0.99] and AUC of the HSROC was 0.97 95% CI [0.95–0.98]; Conclusions: PET/CT has a very high diagnostic accuracy, especially for specificity, to detect LNM in EOC and should be realized systematically, additionally to CT recommended to evaluate peritoneal spread, in the preoperative staging of patients with an advanced disease.

## 1. Introduction

Ovarian cancer is the fifth most common cause of cancer death in the United States, with an estimated 21,570 new cases diagnosed and 13,940 deaths in 2020, and accounts for almost half of all deaths related to pelvic gynecological cancers [1]. Despite the frequency of lymph node metastases (LNM) in epithelial ovarian cancer (EOC) [2], pelvic and para-aortic lymphadenectomy are not part of the cytoreductive surgery in all patients. While lymphadenectomy has to be systematic in the early stage of EOC [3], the randomized LION trial restricted its indication in advanced EOC to patients with suspect lymph nodes before and during surgery because no improvement of survival was observed in the group with systematic lymphadenectomy compared to the group with no lymphadenectomy [4].

Several non-invasive modalities of preoperative imaging are used to assess lymph node status and particularly computed tomography (CT) and positron emission tomography/computed tomography (PET/CT). Advanced EOC is recommended to be radiologically staged with preoperative CT, especially to evaluate peritoneal spread despite its modest diagnostic performance in detecting LNM [3,5]. On the other hand, preoperative PET/CT is not systematically recommended, but its use is increasing probably because it is more accurate to assess lymph node status in other gynecological cancers. Therefore, today, the challenge is to realize the most performant preoperative imaging to detect LNM in order to decide correctly who should or should not have pelvic and para-aortic lymphadenectomy in advanced EOC.

The aim of this systematic review and meta-analysis was to evaluate the diagnostic accuracy of preoperative CT and PET/CT to detect pelvic and para-aortic LNM in patients with EOC.

## 2. Materials and Methods

### 2.1. Design and Registration

This systematic review and meta-analysis have followed the Preferred Reporting Items for Systematic Reviews and Meta-Analyses (PRISMA) guidelines [6]; Figure S1 presents the PRISMA checklist. We have registered the protocol on the PROSPERO international database; information on the protocol is available at http://www.crd.york.ac.uk/prospero/ (accessed on 20 September 2021), no. CRD42020179214. Ethical approval or written informed consent was not necessary.

### 2.2. Search Strategy

A literature search was performed to find relevant published articles about the diagnostic accuracy of preoperative CT and PET/CT to detect pelvic and para-aortic LNM in patients with EOC.

MEDLINE (PubMed), EMBASE, Web of Science and The Cochrane Library databases were systematically explored from January 1990 to May 2021. We restricted our search to English and French languages.

The search was conducted using combinations of the following keywords: ([“ovarian cancer”] OR [“ovarian neoplasm”] OR [“ovarian carcinoma”] OR [“ovarian tumor”] OR [“ovarian tumour”]) AND ([“lymph node”] OR [“nodal”] OR [“lymphadenectomy”] OR [“lymphadenopathy”] OR [“lymphatic”] OR [“paraaortic”] OR [“para-aortic”]) AND ([“accuracy”] OR [“diagnostic value”] OR [“diagnostic performance”] OR [“sensibility”] OR [“specificity”]) AND ([“CT”] OR [“PET/CT”] OR [“imaging”] OR [“radiological”] OR [“radiologic” ] OR [“computed tomography”] OR [“positron emission tomography computed tomography”]).

### 2.3. Eligibility Criteria

Eligibility criteria were as follows: (1) studies assessing the diagnostic accuracy of preoperative CT and PET/CT to detect pelvic and para-aortic LNM in patients with EOC, (2) cytoreductive surgery including pelvic and para-aortic lymphadenectomy or lymph node sampling with histopathological examination of the nodes served as the gold standard, (3) studies reporting or providing sufficient information to calculate the number of true-positive (TP), false-positive (FP), true-negative (TN), false-negative (FN).

The exclusion criteria were as follows: (1) studies focusing on patients with recurrent ovarian cancer, (2) studies focusing on patients with neoadjuvant chemotherapy.

### 2.4. Study Selection

Study selection was independently done by two reviewers (CM and CH). First, possible inclusion was assessed upon title and abstract. Then, if it was suggested relevant, full-text versions were screened to ensure eligibility according to our criteria. Finally, we selected articles to include in the meta-analysis. Any conflicts were resolved by discussion. Duplicates were removed.

### 2.5. Data Extraction and Quality Assessment

One reviewer (CM) recorded data from each selected study with a customized extraction form and a second reviewer (CH) checked all extracted data. The data of interest were collected: author, year and country of publication, study characteristics (study design, number of centers, inclusion interval), inclusion and exclusion criteria (type of imaging, type of cancer included, histology, FIGO stage, surgery), gold standard, preoperative CT and PET/CT protocols, number of patients, patients mean age and information to build 2 × 2 contingency tables (TP, FP, TN and FN).

Two reviewers (CM and CH) independently assessed the risk of bias for each study using the QUADAS-2 tool [7]. QUADAS-2 was performed with Review Manager 5.3.

### 2.6. Statistical Analysis

Bivariate random-effects models [8] was performed to calculate pooled summary estimates of sensitivity, specificity, positive likelihood ratio (LR+), negative likelihood ratio (LR−) and diagnostic odds ratio (DOR) with their 95% confidence intervals (CIs) of preoperative CT and PET/CT to detect pelvic and para-aortic LNM, from the number of TP, FP, FN and TN informed in the studies. A hierarchical summary receiver operator curve (HSROC) was created, by displaying pooled sensitivity and specificity, to obtain the area under the curve (AUC) that reflects the overall accuracy of preoperative CT and PET/CT to detect pelvic and para-aortic LNM [9]. Heterogeneity of the pooled studies was assessed using Cochran’s Q test and I^2^ index (I^2^ > 50% was considered substantial heterogeneity) [10]. Fagan’s nomogram was used to evaluate the clinical utility of preoperative imaging [11].

The “Midas module” for meta-analysis of diagnostic accuracy studies was used in STATA version 13.1 (College Station, TX, USA) [12]. A *p*-value less than 0.05 was considered statistically significant.

## 3. Results

### 3.1. Study Selection

**Figure 1:**
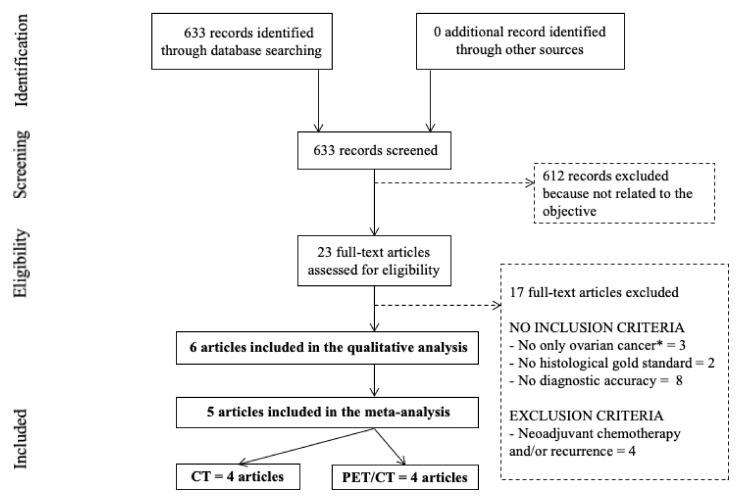
PRISMA flow chart. * borderline ovarian tumors, benign ovarian tumors, other primary cancers. CT: computed tomography; PET/CT: positron emission tomography/computed tomography.

The flow chart of the study selection process is presented in Figure 1. The initial search results generated 633 articles. After screening, based on title and abstract review, 23 articles were assessed for eligibility [13,14,15,16,17,18,19,20,21,22,23,24,25,26,27,28,29,30,31,32,33,34,35]. After reading full-text articles, 5 articles were included in the meta-analysis [13,14,15,16,17,18]. Four of those articles studied the diagnostic accuracy of preoperative CT to detect pelvic and para-aortic LNM [13,14,15,17], and four of those articles studied the diagnostic accuracy of preoperative PET/CT to detect pelvic and para-aortic LNM [13,15,16,17].

Seventeen articles were excluded. Thirteen articles did not respect inclusion criteria: three articles included not only ovarian cancer (but also benign ovarian tumor, borderline ovarian tumor and other primary cancer) [19,20,21], the gold standard was not histology, but surgical findings in two articles [22,23] and diagnostic accuracy of preoperative CT or PET/CT to detect pelvic and para-aortic LNM was not studied in eight articles [24,25,26,27,28,29,30,31]. Four articles presented exclusion criteria: patients included had recurrent ovarian cancer and/or received neoadjuvant chemotherapy [32,33,34,35].

### 3.2. Study Description

The characteristics of studies and participants included in the meta-analysis are summarized in Table 1. Preoperative CT and PET/CT protocols are summarized in Table S1. The studies were all published in English, between 2004 and 2017, and gathered a total of 106 patients for the diagnostic accuracy of preoperative CT to detect pelvic and para-aortic LNM and 138 patients for the diagnostic accuracy of preoperative PET/CT to detect pelvic and para-aortic LNM. They all used histology of lymph nodes as the gold standard with lymphadenectomy or lymph node sampling during primary cytoreductive surgery. All patients had preoperative CT and/or 18-FDG PET/CT within two weeks before surgery.

### 3.3. Quality Assessment

**Figure 2:**
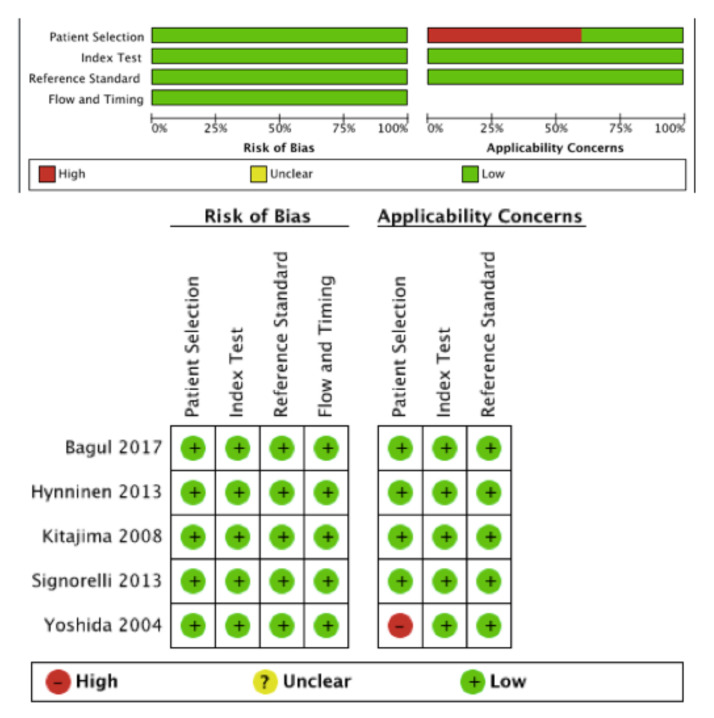
QUADAS-2 risk of bias and applicability concerns.

The methodological quality of studies included in the meta-analysis is illustrated in Figure 2. The quality of the included studies was high. The principal risk of bias interested patient selection in Yoshida study [13]. Indeed, 15 patients were included in this study and one of them did not present an EOC but an ovarian dysgerminoma.

### 3.4. Statistical Analysis

#### 3.4.1. Diagnostic Accuracy

**Figure 3:**
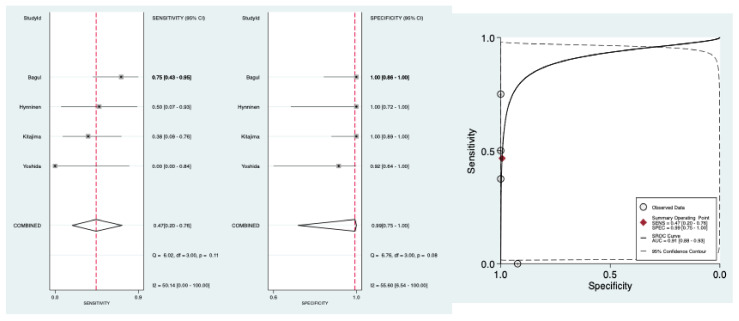
Forest plots of pooled sensitivity and specificity and HSROC of preoperative CT to detect pelvic and para-aortic LNM.

- Preoperative CT:

Table 2 shows pooled results of the diagnostic accuracy of preoperative CT to detect pelvic and para-aortic LNM. The pooled sensitivity was 0.47 95% CI [0.20–0.76], the pooled specificity was 0.99 95% CI [0.75–1.00], the pooled LR− was 0.54 95% CI [0.30–0.98] and the pooled LR+ was 75.40 95% CI [1.20–4611.90]. The forest plots of pooled sensitivity and specificity are exposed in Figure 3. The HSROC curve is presented in Figure 3 and the AUC was 0.91, 95% CI [0.88–0.93]. There was not significant heterogeneity for sensitivity and specificity, respectively: Q = 6.02; *p* = 0.11; I^2^ = 50.14, 95% CI [0.00–100.00] and Q = 6.76; *p* = 0.08; I^2^ = 55.60, 95% CI [6.54–100.00] (Figure 3).

**Figure 4:**
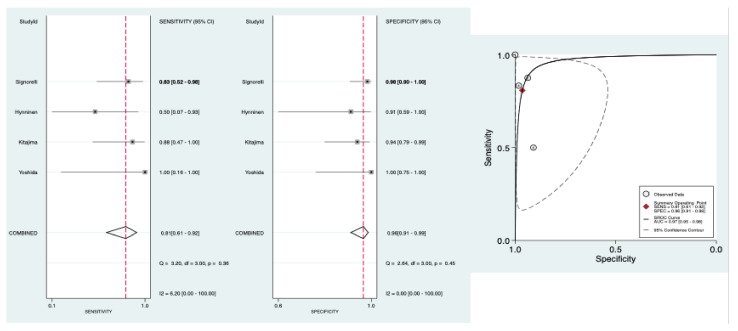
Forest plots of pooled sensitivity and specificity and HSROC of preoperative PET/CT to detect pelvic and para-aortic LNM.

- Preoperative PET/CT:

Table 2 shows pooled results of the diagnostic accuracy of preoperative PET/CT to detect pelvic and para-aortic LNM. The pooled sensitivity was 0.81 95% CI [0.61–0.92], the pooled specificity was 0.96 95% CI [0.91–0.99], the pooled LR− was 0.20 95% CI [0.09–0.44] and the pooled LR+ was 22.60 95% CI [8.50–60.30]. The forest plots of pooled sensitivity and specificity are exposed in Figure 4. The HSROC curve is presented in Figure 4 and the AUC was 0.97, 95% CI [0.95–0.98]. There was not significant heterogeneity for sensitivity and specificity, respectively: Q = 3.20; *p* = 0.36; I^2^ = 6.20, 95% CI [0.00–100.00] and Q = 2.64; *p* = 0.45; I^2^ = 0.00, 95% CI [0.00–100.00] (Figure 4).

#### 3.4.2. Publication Bias

Deek’s funnel plot asymmetry test did not reveal evidence of publication bias for preoperative CT studies (*p* = 0.07) nor preoperative PET/CT studies (*p* = 0.29) (Figures S2 and S3).

#### 3.4.3. Clinical Utility

**Figure 5:**
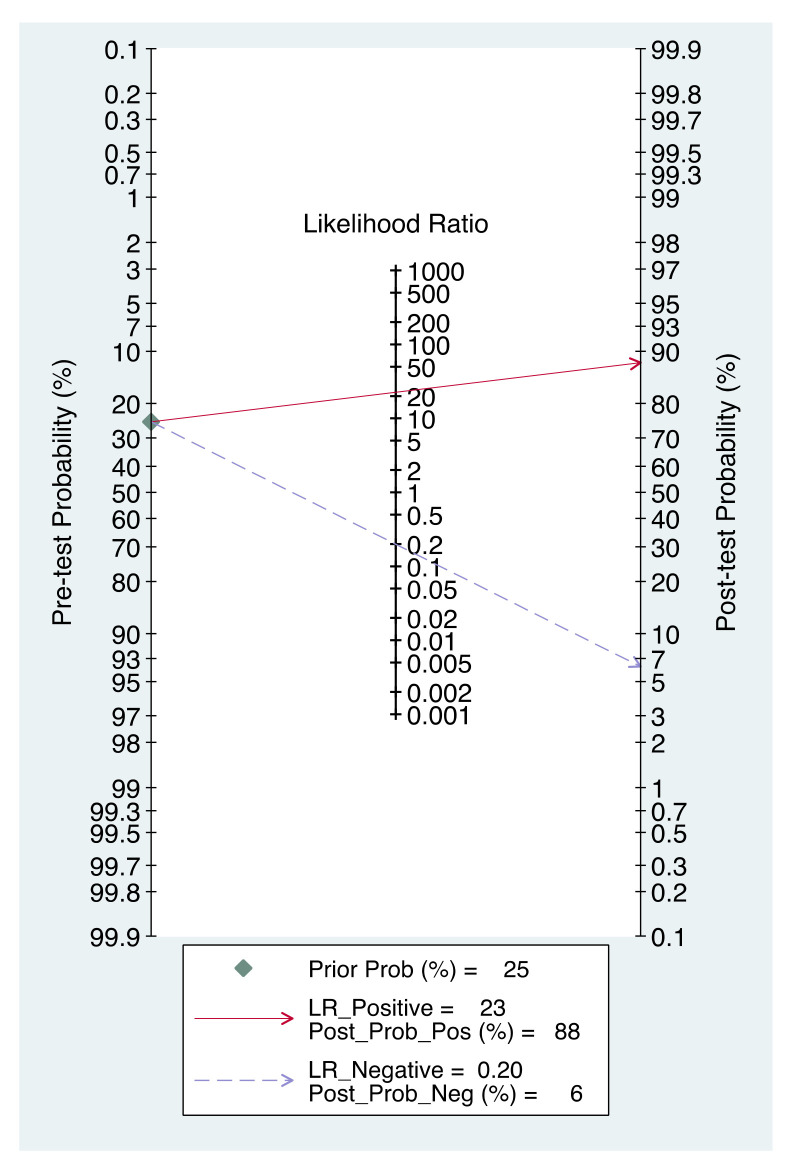
Fagan’s nomogram for likelihood ratios and the probability of preoperative PET/CT for detecting lymph node metastases.

Figure 5 shows Fagan’s nomogram of preoperative PET/CT for likelihood ratios. The LNM pre-test probability was 25%. The nomogram indicated that positive preoperative PET/CT increased the LNM post-test probability to 88% and that negative preoperative PET/CT reduced the LNM post-test probability to 6%. Figure S4 shows Fagan’s nomogram of preoperative CT for likelihood ratios.

## 4. Discussion

Additionally, to evaluate peritoneal spread to judge if complete cytoreductive surgery is feasible, CT and PET/CT are used to assess lymph node status in order to decide if lymphadenectomy should be performed during this surgery (presence of suspect lymph nodes) or should not be performed (no suspect lymph node). We conducted the first meta-analysis that evaluates the diagnostic accuracy of preoperative CT and PET/CT to detect pelvic and para-aortic LNM in EOC. For preoperative CT, four articles were included involving, 106 patients; pooled sensitivity was 0.47 95% CI [0.20–0.76], pooled specificity was 0.99 95% CI [0.75–1.00] AUC HSROC was 0.91 95% CI [0.88–0.93]. For preoperative PET/CT, four articles were included, involving 138 patients; pooled sensitivity was 0.81 95% CI [0.61–0.92], pooled specificity was 0.96 95% CI [0.91–0.99] and AUC of the HSROC was 0.97 95% CI [0.95–0.98].

This meta-analysis has many strengths. We observed a standardized protocol with a comprehensive search strategy, study selection, and data extraction. All studies included in the meta-analysis were prospective. The quality of the five included studies showed a low risk of bias for the four domains (patient selection, index test, reference standard, flow, and timing); in particular, all the LNM were confirmed histologically and permitted a misclassification bias to be excluded. We included only studies with primary cytoreductive surgery without neoadjuvant chemotherapy that could have histologically sterilized LNM and result in misclassifications bias. Bivariate random-effects models and HSROC curves were performed; the patient samples were pooled so that the findings of this meta-analysis are more robust than any of the individual studies.

This meta-analysis also has limitations. First, only five studies with few patients were included because the literature is poor and our inclusion criteria were severe. In addition, we note that none of those studies were randomized control trials. Indeed, two studies were excluded because the gold standard for LNM diagnosis was surgical findings; even if intraoperative clinical examination of lymph node has good accuracy, it cannot be considered as histological examination [36]. Moreover, three studies were excluded because they included suspicions of ovarian cancer that were not confirmed at final histology. Then, we decided to include not only studies concerning advanced EOC but also studies with early-stage EOC; however, we think it did not impact the results of our meta-analysis since all patients had preoperative imaging and cytoreductive surgery with lymphadenectomy or lymph node sampling. Finally, one out of the five studies of the meta-analysis included not only EOC but also one dysgerminoma [13]; however it was, only one patient out of the 15 patients included in this study.

Other preoperative imaging exists to assess lymph node status before surgery: PET/CT with another tracer than 18-FDG and magnetic resonance imaging (MRI). We found no study that evaluated the diagnostic accuracy of PET/CT with another tracer than 18-FDG to detect LNM in patients with EOC and only one for MRI: sensibility and specificity were respectively 62.5% and 86.7% [37].

As mentioned above, in the post-LION era, lymph node status has to be assessed before and during cytoreductive surgery in order to decide if lymphadenectomy should be performed (presence of suspect lymph nodes) or should not be performed (no suspect lymph node) in advanced EOC [4]:

- Before surgery, savant societies recommended a preoperative CT, mostly because of its high accuracy to evaluate peritoneal spread, despite the necessity to assess lymph node status as accurately as possible to not impact patient prognosis [3,5]. Our meta-analysis, is clearly in favor of preoperative PET/CT for detecting pelvic and para-aortic LNM; even if we could define a group at high risk of LNM for patients with positive preoperative CT and PET/CT (LR+ > 4 and specificity > 90%) associated with a small number of FP, we could only define a group at low risk of LNM for patients with negative PET/CT (LR− < 0.25 and sensibility > 95%) [38]. Indeed, preoperative CT, with its low sensitivity and high LR−, is associated with a high number of FN and is not sufficient to conclusively rule out LNM. This is highlighted in Figure 5: when negative preoperative PET/CT reduces LNM probability from 25% to 6%, negative preoperative CT reduces it only to 15%. The study of Choi and al. that could not be included in the meta-analysis because its report lacked data to build a complete contingency 2 × 2 table found similar results for preoperative CT with a very low sensibility of 24% [18]. In locally advanced cervical cancer, comparable findings were made, and so two preoperative imaging are now recommended: magnetic resonance imaging (MRI) for assessment of pelvic tumor extent but also PET/CT for assessment of nodal disease since para-aortic lymphadenectomy depends on the lymph node status [39].
- During surgery, LION study recommends an intraoperative clinical examination (IOCE) of lymph node. In a previous meta-analysis, including five studies, we evaluated the diagnostic accuracy of IOCE for detecting pelvic and para-aortic LNM. Once again, preoperative PET/CT seems to be superior to IOCE to eliminate LNM with a higher sensibility and a lower LR−: 0.81 95% CI [0.61–0.92] and 0.20 95% CI [0.09–0.44] vs 0.79 95% CI [0.67–0.87] and 0.25 95% CI [0.16–0.38] respectively [36].

In conclusion, this meta-analysis demonstrates the high diagnostic accuracy of preoperative PET/CT, especially for specificity, to detect pelvic and para-aortic LNM in EOC. Consequently, PET/CT should be done, additionally to CT recommended to evaluate peritoneal spread and to IOCE, systematically in the preoperative staging of advanced EOC.

## Figures and Tables

**Table 1:** Studies and participant characteristics included in the quantitative analysis.

Study, Year, Country	Design of Study	Number of Center	Inclusion Interval	CT and/or PET/CT	Gold Standard	Number of Patients	Median Age (years)	Type of Cancer	Histology	FIGO Stage	Surgery
Yoshida, 2004, Japan [13]	Prospective	One	From September 2001 to July 2002	CT and 18F-FDG PET/CT	Histology	15	-	Ovarian cancer	Epithelial adenocarcinoma - Serous = 8 - Mucinous = 3 - Endometrioid = 2 - Undifferentiated = 1 - Dysgerminoma = 1	IA = 2 IC = 3 IIB = 1 IIC = 2 IIIB = 1IIIC = 6	Primary Laparotomy cytoreductive surgery Lymphadenectomy or lymph node sampling
Bagul, 2017, India [14]	Prospective	One	From March 2013 to May 2015	CT	Histology	36	51 (range, 39–74)	Ovarian and peritoneal cancer	Epithelial adenocarcinoma - Serous = 20 - Mucinous = 1 - Endometrioid = 2 - Clear cell = 3 - Undifferentiated = 10	IIIC	Primary Laparotomy cytoreductive surgery Lymphadenectomy
Hynninen, 2013, Finland [15]	Prospective	One	From October 2009 to March 2012	CT and 18F-FDG PET/CT	Histology	15	65 (range, 45–79)	Ovarian, fallopian and peritoneal cancer	Epithelial adenocarcinoma	III and IV	Primary Laparotomy cytoreductive surgery Lymphadenectomy or lymph node sampling
Signorelli, 2013, Italy [16]	Prospective	One	From 2006 to 2012	18F-FDG PET/CT	Histology	68	49 (range, 35–72)	Ovarian cancer	Epithelial adenocarcinoma - Serous = 29 - Mucinous = 6 - Endometrioid = 10 - Clear cell = 10 - TNMM = 3 - Mixed = 7 - Undifferentiated = 3	IA = 7 IB = 2 IC = 27 IIA = 2 IIB = 11 IIC = 3 IIIA = 4 IIIC = 12	Primary Laparotomy cytoreductive surgery Lymphadenectomy
Kitajima, 2008, Japan [17]	Prospective	One	From April 2006 to April 2008	CT and 18F-FDG PET/CT	Histology	40	55.4 (range, 38–77)	Ovarian cancer	Epithelial adenocarcinoma - Papillary serous = 11 - Serous = 4 - Mucinous = 7 - Endometrioid = 5 - Clear cell = 7 - Undifferentiated = 6	IA = 9 IB = 3 IC = 6 IIA = 2 IIB = 3 IIC = 2 IIIA = 1 IIIB = 3 IIIC = 10 IV = 1	Primary Laparotomy cytoreductive surgery Lymphadenectomy

CT: computed tomography; PET/CT: Positron emission tomography/computed tomography.

**Table 2:** Diagnostic accuracy of preoperative CT and PET/CT for detecting lymph node metastases.

Study	TP	FP	FN	TN	Pooled Sensitivity 95% CI	Pooled Specificity 95% CI	Pooled LR+ 95% CI	Pooled LR− 95% CI	AUC 95% CI	*p*-Value of Deek’s Funnel Plot
CT										
Yoshida	0	1	2	12	0.47 0.20–0.76	0.99 0.75–1.00	75.40 1.20–4611.90	0.54 0.30–0.98	0.91 0.88–0.93	0.07
Kitajima	3	0	5	32						
Hynninen	2	0	2	11						
Bagul	9	0	3	24						
PET/CT										
Yoshida	2	0	0	13	0.81 0.61–0.92	0.96 0.91–0.99	22.60 8.50–60.30	0.20 0.09–0.44	0.97 0.95–0.98	0.29
Kitajima	7	2	1	30						
Hynninen	2	1	2	10						
Signorelli	10	1	2	55						

TP: true positive; FP: false positive; TN: true negative; FN: false negative; FN: false negative; CI: confidence interval; LR: likelihood ratio; AUC: area under the curve.

## Supplementary Materials

The following are available online at https://www.mdpi.com/article/10.3390/diagnostics11101748/s1, Figure S1: PRISMA checklist, Table S1: Preoperative CT and PET/CT protocols, Figure S2: Deek’s funnel plot asymmetry test of preoperative CT for detecting lymph node metastases, Figure S3: Deek’s funnel plot asymmetry test of preoperative PET/CT for detecting lymph node metastases, Figure S4: Fagan’s nomogram for likelihood ratios and the probability of preoperative CT for detecting lymph node metastases.
