# Supplementary material for: Preoperative CT or PET/CT to Assess Pelvic and Para-Aortic Lymph Node Status in Epithelial Ovarian Cancer? A Systematic Review and Meta-Analysis
Source: Diagnostics (Basel). 2021 Sep 23;11(10):1748. doi: 10.3390/diagnostics11101748 (PMC8534764; doi:10.3390/diagnostics11101748)
Supplement: Supplementary file 1 [file diagnostics-11-01748-s001.zip › diagnostics-1353248-supplementary.pdf]

Section/topic	#	Checklist item
Risk of bias across studies	15	Specify any assessment of risk of bias that may affect the cumulative evidence (e.g., publication bias, selective reporting within studies).
Additional analyses	16	Describe methods of additional analyses (e.g., sensitivity or subgroup analyses, meta-regression), if done, indicating which were pre-specified.
**RESULTS**		
Study selection	17	Give numbers of studies screened, assessed for eligibility, and included in the review, with reasons for exclusions at each stage, ideally with a flow diagram.
Study characteristics	18	For each study, present characteristics for which data were extracted (e.g., study size, PICOS, follow-up period) and provide the citations.
Risk of bias within studies	19	Present data on risk of bias of each study and, if available, any outcome level assessment (see item 12).
Results of individual studies	20	For all outcomes considered (benefits or harms), present, for each study: (a) simple summary data for each intervention group (b) effect estimates and confidence intervals, ideally with a forest plot.
Synthesis of results	21	Present results of each meta-analysis done, including confidence intervals and measures of consistency.
Risk of bias across studies	22	Present results of any assessment of risk of bias across studies (see Item 15).
Additional analysis	23	Give results of additional analyses, if done (e.g., sensitivity or subgroup analyses, meta-regression [see Item 16]).
**DISCUSSION**		
Summary of evidence	24	Summarize the main findings including the strength of evidence for each main outcome; consider their relevance to key groups (e.g., healthcare providers, users, and policy makers).
Limitations	25	Discuss limitations at study and outcome level (e.g., risk of bias), and at review-level (e.g., incomplete retrieval of identified research, reporting bias).
Conclusions	26	Provide a general interpretation of the results in the context of other evidence, and implications for future research.
**FUNDING**		
Funding	27	Describe sources of funding for the systematic review and other support (e.g., supply of data); role of funders for the systematic review.

Figure S1. PRISMA checklist.

Table S1. Preoperative CT and PET/CT protocols.

Study	Radiologist protocol	Imagery protocol		
		Delay before surgery	LN location and LNM diagnosis	Technique
**CT and PET/CT**				
**Yoshida**	Before surgery, the results of diagnostic imaging were evaluated for each patient (CT with FDG-PET) by the reviewers who comprised one nuclear medicine specialist, one radiologist, and two other gynecologist oncologists who had experience with these methods and no knowledge of patient identity at the time of evaluation; conclusions arrived at by consensus of the reviewers	2 weeks maximum	- Aortic LN<br><br>- CT: LN > 1cm<br><br>PET/CT: Hypermetabolic LN	CT: CT was performed using a helical CT scanner (HiSpeed Advantage, General Electric Yokogawa Medical Systems, Tokyo, Japan). Both unenhanced and contrast-enhanced scanning were performed. For contrast-enhanced imaging, IV administration of 100 mL of contrast material via a power injector at a rate of 1 mL/sec was performed.<br><br>PET/CT: All subjects underwent PET using an Advance scanner (General Electric Medical Systems, Milwau- kee, WI), which permits simultaneous acquisition of 35 image slices with interslice spacing of 4.25 mm. Performance tests showed intrinsic resolution of 4.6-5.7 and 4.0-5.3 mm in the transaxial and axial directions, respectively. FDG was produced using a small cyclotron (OSCAR3, Oxford Instruments, Oxford, UK) and an automated synthesis system (NKK, Tokyo, Japan). After the patients had fasted for at least 12 hr before radiotracer administration, approximately 370 MBq of FDG was injected IV. Whole-body emission scanning was started 40–60 min after FDG administration, and PET data were acquired for 12-14 min with six or seven bed positions. A transmission scan was obtained using a germanium-68–gallium-68 rod source for attenuation correction after the emission scan at the same bed positions as the emission scan. The PET data were reconstructed using the iterative reconstruction algorithm and segmented attenuation correction (IRA/SAC) method and resliced into transaxial, coronal, and sagittal sections with the gray scale in the standardized uptake values. PET findings for the transaxial, coronal, and sagittal sections were interpreted.
**Kitajima**	CT: Enhanced full-dose CT component images were retrospectively evaluated in consensus by two experienced radiologists (readers A and B with 8 and 20 years of experience in CT, respectively) who had knowledge of neither the other imaging results nor the clinical data<br><br>PET/CT: Integrated FDG-PET/contrast-enhaced CT images were retrospectively interpreted	2 weeks maximum	- Aortic LN<br><br>- CT: LN > 1cm and/or the presence of a central unenhanced area suggesting central necrosis were considered a sign of malignancy; the presence of peripheral low attenuation suggesting a fatty hilum was considered a benign sign<br><br>PET/CT: LN with increased glucose uptake were deemed positive for metastatic spread, even if they were smaller than 1cm;	Whole-body imaging was performed using a combined PET/CT scanner (Biograph, Sensation 16 PET/CT, Siemens AG, Erlangen, Germany). CT covered a region ranging from the meatus of the ear to the mid-thigh. The technical parameters of the 16-detector row helical CT were a gantry rotation speed of 0.5 s, a table speed of 24 mm per gantry rotation. The PET component of the combined imaging system had an axial view of 16.2 cm (per bed position) with an interslice spacing of 3.75 mm in one bed position and provided an image from the meatus of the ear to the mid-thigh with six to seven bed positions. The transaxial field of view and pixel size of the PET images reconstructed for fusion were 58.5 cm and 4.57 mm, respectively, with a matrix size of 128Å~128. To avoid artifacts caused by the urinary tract, patients were asked to drink 1,000 ml of water 1-2 h prior to image acquisition, and to void just before the start of acquisition. No urinary bladder catheterization was used. After at least 4 h of fasting, patients received an intravenous injection of 4.0 MBq/kg body weight of FDG. The blood glucose levels were checked in all patients before FDG injection,

	in consensus by two experienced radiologists (readers C and D with 3 and 5 years of experience in PET/CT, respectively) who had knowledge of neither the other imaging results nor the clinical data		conversely, LN with no detectable tracer uptake were deemed negative for metastatic spread, even if they were larger than 1cm	and no patients showed a blood glucose level of more than 160 mg/dl. About 50 min later, initially unenhanced low-dose CT was performed at 140 kV and 40 mA for attenuation correction of PET image. A whole-body emission PET/CT was performed immediately after the low-dose CT, with a 3-min acquisition per bed position using a threedimensional acquisition mode. Attenuation-corrected PET images were reconstructed with an ordered-subset expectation maximization iterative reconstruction algorithm (eight subsets, three iterations). Finally, diagnostic contrast- enhanced full-dose CT was performed for the same axial coverage at 140 kVand 230 mA, with 2-mm slice thickness. Intravenous administration of a total volume of 150 mL (maximum) or 2 mL/kg of iodinated contrast material (Iomeprole 300, Eisai, Tokyo, Japan) containing 300 mg of iodine per milliliter via power injection at a rate of 2.5 mL/s was performed, and the scan of neck~thorax, upper~middle abdomen, and lower abdomen~pelvis was started at 45, 75, and 90 s, respectively, after injection. Oral contrast agent was not administered. PET, CT, and fused PET/CT images were generated for review on a computer workstation (AZE Virtual Place Version 3.0035).
**Hynninen**	CT: Whole body contrast-enhanced CT images were evaluated by two experienced radiologists, who were not aware of the PET/CT results or findings at surgery<br><br>PET/CT: Integrated FDG-PET/high dose contrast-enhanced CT images were interpreted by two experienced nuclear medicine physicians blinded to the surgical findings	2 weeks maximum	- Pelvic and aortic LN<br><br>- CT: -<br><br>PET/CT: LN with increased glucose uptake were considered positive for metastatic spread regardless of size	Preoperative whole-body contrast-enhanced FDG PET/CT (64-row Discovery STE or VCT, General Electric Medical Systems, Milwaukee, WI, USA) was performed. All patients fasted for a minimum of 6 h and their serum glucose was controlled before the intravenous injection of 4 Mbq/kg 18F-FDG. After 50-60 min from injection low-dose PET/CT (kV 120, Smart mA range 10-80) from skull base to mid-thigh was followed with whole-body diagnostic high dose contrast-enhanced CT scan (kV 120, Smart mA range100-440) after automated intravenous injection of contrast agent. PET images were reconstructed with 128 Å~ 128 matrix size in fully 3D mode using ML-OSEM reconstruction algorithm. Imaging analysis was performed using ADW 4.5 workstation.
**CT only**				
**Bagul**	All the preoperative CT were systematically reviewed by the assigned radiologists before surgery who were blinded for the clinical decision of treatment	2 weeks maximum	- Pelvic and aortic LN<br><br>- LN > 1cm	CT were performed with a high-speed scanner—Siemens Somatom Sensation 64-slice MDCT (Siemens Medical Systems, Iselin, NJ). After oral administration, 40 ml of 2% Gastrografin contrast in 2 l of water, a CT was obtained at baseline and 30 s after intravenous administration of 80 ml of nonionic contrast at 2.3ml/s rate. Images were obtained with 3-5 mm thickness and 15 mm/s table speed.
**PET/CT only**				
**Signorelli**	Images were evaluated by two nuclear medicine physicians informed about the clinical data of patients at the moment of the CT	-	- Pelvic and aortic LN<br><br>- Presence of focal increased tracer uptake, regardless of their size on CT; LN with no detectable tracer uptake were reported as benign	All studies were performed with a PET/CT (Discovery ST or Discovery 600-GE Healthcare, Milwaukee, WI, USA) consisting of a PET scanner and a multi-detector CT scanner, which allows the acquisition of co-registered CT and PET images from the same patient in one session. Patients fasted for at least 6 h before the intravenous administration of 300-370 MBq of 18F-FDG. Patients with blood glucose levels above 170 mg/dL were excluded from the study. In addition, all patients were orally hydrated (500 mL of water) during the FDG

	uptake period and were asked to empty their bladder before positioning for the scan. The combined examination was started about 60 min after the FDG injection. CT was acquired first, during shallow breathing, with 140 kV, 60 mA and 3.75 mm of slice thickness. No oral contrast or intravenous contrast was used for the CT component of the examination. PET was acquired in 3D mode with 3 min in each bed position; PET images were reconstructed with iterative algorithm (ordered subset expectation maximization - OSEM), 128 Å~ 128 (Discovery ST) or 256x256 (Discovery 600) matrix size, attenuation, random and scatter correction. Attenuation correction was performed on the basis of CT data. The CT pixel values, which were measured in Hounsfield units, were transformed into linear attenuation coefficients for the 511-keV energy radiation. Image analysis was performed as follows: attenuation-corrected PET images, CT images and co-registered PET/CT images were displayed together on the workstation (Xeleris, AW4.4 GE Healthcare, Milwaukee, WI, USA).

CT: computed tomography; PET/CT: positron emission tomography/computed tomography; LN: lymph node; LNM: lymph node metastasis; FDG: fluorodeoxyglucose; MDCT: multidetector CT

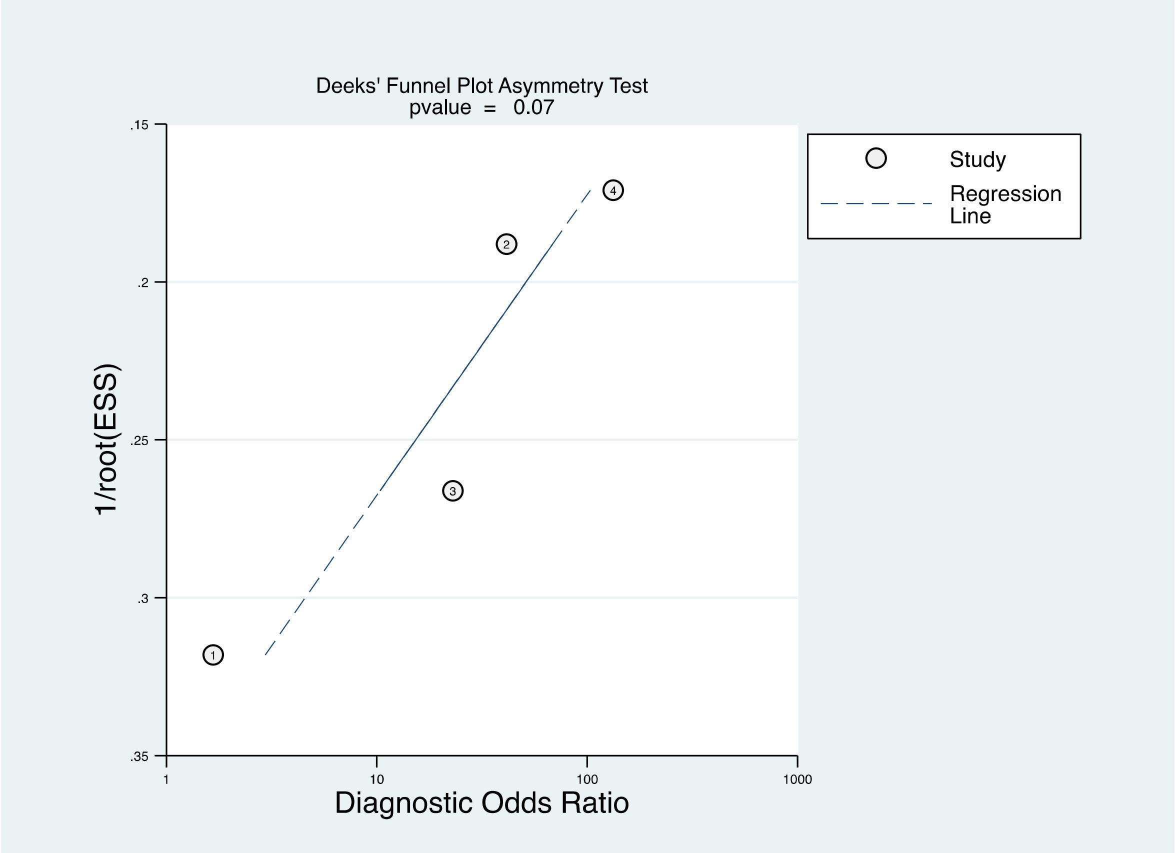

Figure S2. Deek's funnel plot asymmetry test of preoperative CT for detecting lymph node metastases. 1: Bagul; 2: Hynninen; 3: Eltabbakh; 4: Kitajima; 5: Yoshida; ESS: effectif sample size.

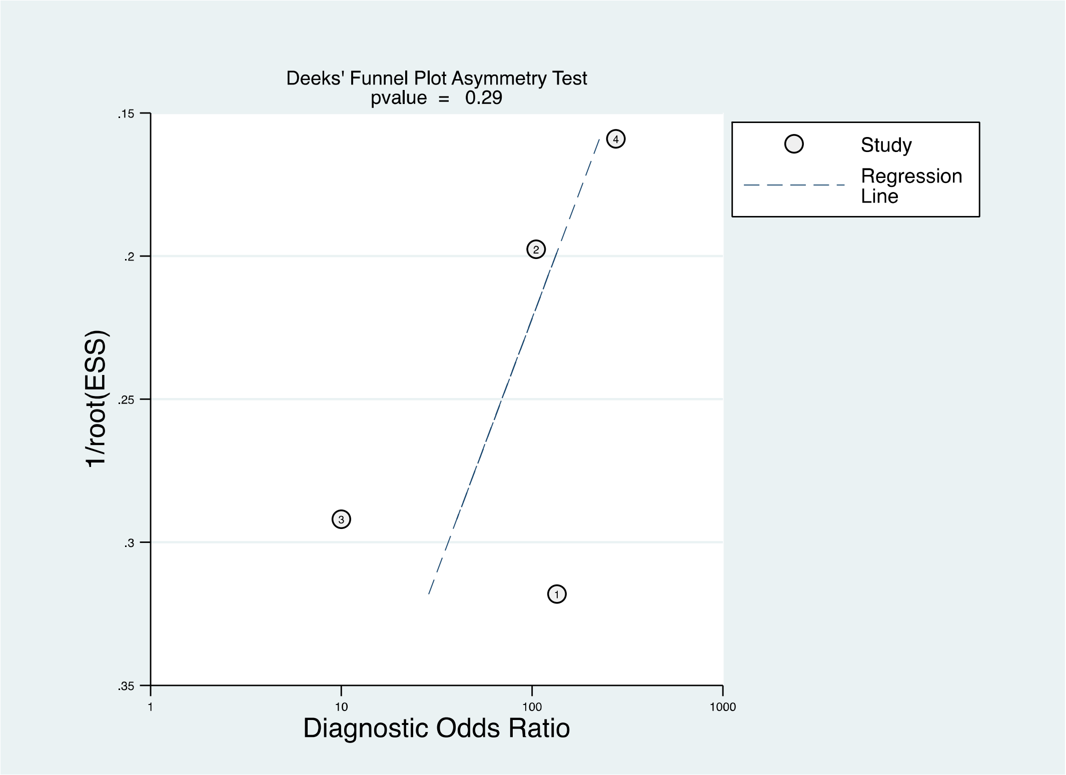

Figure S3. Deek's funnel plot asymmetry test of preoperative PET/CT for detecting lymph node metastases. 1: Signorelli; 2: Hynninen; 3: Eltabbakh; 4: Kitajima; 5: Yoshida; ESS: effectif sample size.

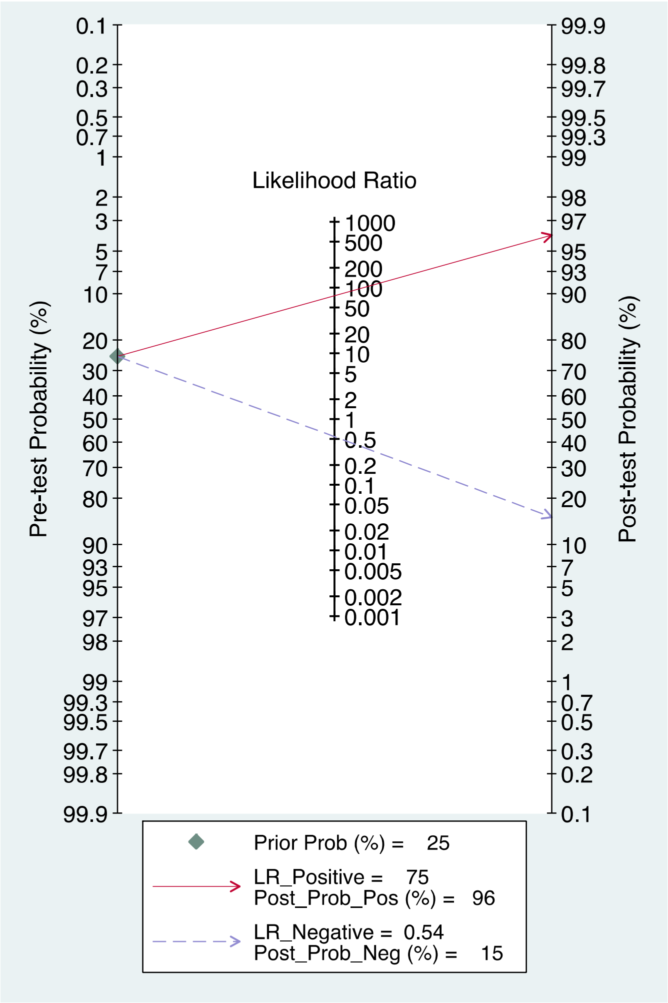

Figure S4. Fagan's nomogram for likelihood ratios and the probability of preoperative CT for detecting lymph node metastases.
